# A Suggestion: Letter from Dr. Bell

**Published:** 1900-07

**Authors:** James Hall Bell

**Affiliations:** San Antonio, Texas


### A Suggestion: Letter From Dr. Bell.

San Antonio, Texas, June 1, 1900.

*Editors of Texas Medical Journal:*

In the early part of the present year I prepared a paper for presentation to the Texas State Medical Association, in which I called the attention of the profession to the eccentric curriculum of the New York Medical College, of San Antonio (the establishment was then graduating students of both sexes and colors in from twelve to seventeen days from date of matriculation), and suggested the urgent necessity of an educational movement among the people at large in behalf of a higher standard of medical culture. The amazing innovations proposed and successfully introduced by this so-called school of medicine were so manifestly fraudulent in character that the Attorney-General of the State was finally induced to institute suit for the forfeiture of its charter. When the case came to trial in the Thirty-seventh District Court, Judge Green presiding, judgment was taken by default by the State, and an injunction issued restraining the infamous institution from issuing, granting or recording diplomas. But the laws that permitted its existence are still in force, and the door-way to the practice of medicine in Texas remains wide open.

Under the provisions of the Revised Statutes of Texas, a private corporation may be created by the voluntary association of three or more persons for the support of any literary, scientific or educational undertaking (R. S., Art. 642, Secs. 2 and 3), and the Supreme Court of Texas has declared in the case of Wilson vs. Vick, and since reaffirmed, that the graduate of any school of medicine chartered by the State is entitled to practice without further legal formality of any kind whatsoever. This means, in effect, that the graduates of the defunct New York Medical College, of San Antonio, shall be allowed to practice medicine in Texas. It means, moreover, that any trio of scoundrels or imbeciles may, with blind incomprehensiveness or indifference to the sacred rights of humanity establish a school of medicine and graduate the most abandoned set of scamps and ignoramuses that ever stumbled head-long into the ranks of a profession in the practice of which "the greatest men have trembled and the wisest erred."

The State Legislature will not hesitate to pass a bill appropriating money for the study, prevention and cure of disease among cattle, but it has never passed a bill which recognizes the duties, the objects, desires and achievements of scientific medicine; never disarmed and suppressed quackery; never exercised its powers to protect the hallowed precincts of the sick-room from the effrontery and mischievousness of legalized pretence, wrong-doing and incapacity. And it never will do anything in the right direction for State medicine until it is forced to it by an aroused public sentiment.

Some years ago, the medical society of the State of New York took up this question, and went to the bottom of it, by proposing the abolition of the power of medical schools in the State of New York to "continue their diplomas as licenses to practice." To lessen opposition, the differences in "schools" were ignored, and the Homeopathic Medical Society of the State of New York were invited to join in the movement. Finally a law was passed by the legislature of that State abrogating the right of the medical schools of New York to license graduates, and this power exclusively given to the State Board of Medical Examiners, which was appointed by the regents of the State, a body representing the three recognized schools of medicine. This salutary law further required that the graduates and licentiates of other States and countries should not be allowed to practice in the State of New York without passing the examination of the State board.

Is the time not opportune, in view of the increasing difficulties of the situation in this State, to harmonize the conflicting interests of opposing schools, and to attempt, by a joint effort, the passage of a bill which shall embody the main features of the law now in force in the State of New York? I, for one, say "yes." For notwithstanding its objections and the incidental hardships imposed by some of its provisions, the New York law is, in my judgment, the wisest and most effective measure ever proposed in this country for the better government of State medicine.

JAMES HALL BELL.

